# Clinical and Biological Significance of a Necroptosis-Related Gene Signature in Glioma

**DOI:** 10.3389/fonc.2022.855434

**Published:** 2022-06-02

**Authors:** Zunjie Zhou, Jing Xu, Ning Huang, Jun Tang, Ping Ma, Yuan Cheng

**Affiliations:** Department of Neurosurgery, The Second Affiliated Hospital of Chongqing Medical University, Chongqing, China

**Keywords:** necroptosis, prognosis, gliomas, tumor microenvironment, signature, immune infiltration

## Abstract

**Background:**

As a novel form of programmed cell death, necroptosis is related to multiple tumor types and their immune microenvironments. However, its association with glioma has not been clarified.

**Methods:**

Necroptosis genes were obtained from the Gene Set Enrichment Analysis (GSEA) database. RNA-seq and clinical data were downloaded from TCGA and CGGA databases. A necroptosis gene signature was constructed based on univariate and multivariate Cox regression analyses. Next, survival analysis, independent prognostic analysis, and nomogram were performed to assess and verify the model. Subsequently, we analyzed the tumor microenvironment (TME) and immune cell infiltration *via* ESTIMATE and CIBERSORTx algorithms. Finally, the response of glioma patients in the TCGA database to immune checkpoint inhibitor (ICI) therapy was predicted using the Tumor Immune Dysfunction and Exclusion (TIDE) database.

**Results:**

Of the seven prognostic necroptosis genes, RIPK1, RIPK3, FAS, and FADD were used to construct the risk signature that accurately predicts the prognosis of glioma patients. Functional enrichment results suggest that necroptosis is correlated with immune response and angiogenesis. Immune analysis revealed that necroptosis can boost inflammatory activity and attract immunosuppressive cell infiltration to form a chronic inflammatory microenvironment, promoting glioma growth. Additionally, glioma patients in the TCGA cohort with high necroptosis gene expression exhibited a better response to ICI therapy predicted by the TIDE algorithm.

**Conclusion:**

We constructed a necroptosis gene signature, which has the potential for use as a biomarker for predicting glioma patients’ prognosis, revealing the association between necroptosis and the immune microenvironment, and serving as a reference for immune therapy.

## Introduction

Gliomas are common malignant craniocerebral tumors of the central nervous system which are characterized by invasiveness, recurrence, malignancy, and poor prognosis (1). Gliomas can be classified as low-grade and high-grade gliomas based on the World Health Organization (WHO) classification scheme (2). Although low-grade gliomas have a better prognosis than high-grade gliomas, greater than half of LGG cases evolve and progress to high-grade glioma after surgery and chemotherapy. Regarding the heterogeneity of gliomas (3), some patients with the same tumor grades exhibit significantly different survival times and therapeutic responses. Although several biomarkers, including IDH mutation status, 1p19q codeletion, and MGMT methylation (4, 5) were included in the 2016 WHO classification to reveal the histological features and guide the therapeutic strategy, these widely used biomarkers cannot accurately predict the prognosis of glioma patients and do not explain the significant differences noted among these patients with the same tumor grades. Thus, the identification of significant and accurate glioma biomarkers is critical for improved diagnosis and treatment.

In addition to autophagy, apoptosis, and pyroptosis, necroptosis is a novel style of programmed cell death that is mainly mediated by activating receptor-interacting protein kinase 1 (RIPK1), RIPK3, and mixed lineage kinase domain-like (MLKL) (6). Initially, necroptosis was observed in infections (7), alcoholic and drug-induced liver injury (8), and spinal cord injury (9). However, an increasing number of studies have shown that it plays a complex role in the development and progression of cancer (10). The expression level of necroptosis genes is decreased in most cancer and is associated with poor prognosis (11). Studies have shown that promoting necroptosis can effectively inhibit tumor growth (12–15). In addition, increasing evidence suggests that as an inflammatory necrosis pathway necroptosis potentially promotes the migration and invasion of some tumors (16–18). However, the mechanism of necroptosis and its prognostic value in glioma remain unclear.

In the present study, a large cohort of primary glioma patients from the TCGA database and normal control cerebral tissues samples from the GTEX database were employed and screened to determine the expression levels of necroptosis genes in gliomas and controls. The risk signature was constructed based on these genes after univariate and multivariate Cox analysis and validated by the Chinese Glioma Genome Atlas (CGGA). Subsequently, the ESTIMATE, CIBERSORT, TIMER2.0, and TIDE algorithms were used to evaluate and clarify the correlation of the risk signature with the tumor immune microenvironment.

## Materials and Methods

### Data Collection and Preprocessing

RNA-seq data of primary gliomas (TCGA-LGG and TCGA-GBM) from The Cancer Genome Atlas (TCGA) database (https://www.cancer.gov/) and normal cerebral tissues from the Genotype-Tissue Expression (GTEX) project (https://www.gtexportal.org/) were downloaded from the UCSC Xena (https://xena.ucsc.edu/) database as the TPM data type. The Sequencing and Array datasets (CGGA-693, CGGA-325, and CGGA-301) were downloaded from the CGGA database (http://www.cgga.org.cn/) as validated cohorts. The sequencing data from CGGA were converted into the TPM data type to correct the effect of transcription sequencing depth and gene length. All the data were screened with criteria of completed survival information and survival times >30 (overall survival <30 was considered that glioma is not the critical factor for the short survival). Finally, we used the “sva” R package to adjust the batch between different cohorts and transformed “log2 (TPM+1)” for the follow-up analysis. The clinical information of three cohorts were showed in the **Table 1**. Additionally, the protein expression levels of necroptosis-related genes between normal cerebral and glioblastoma tissues were obtained from the University of Alabama at Birmingham, cancer data analysis is portal database (UALCAN, Ualcan.path.uab.edu/analysis).

**Table 1:** The clinical characters of different cohorts.

Characteristics	TCGA cohort (N=589)	CGGA_Seq cohort (N=624)	CGGA_Array cohort (N=249)
Age			
<=41	248 (16.96%)	271 (18.54%)	116 (7.93%)
>41	341 (23.32%)	352 (24.08%)	131 (8.96%)
NA	0 (0%)	1 (0.07%)	2 (0.14%)
Gender			
Female	255 (17.44%)	256 (17.51%)	103 (7.05%)
Male	334 (22.85%)	368 (25.17%)	146 (9.99%)
Grade			
G2 (WHO II)	223 (15.25%)	220 (15.05%)	103 (7.05%)
G3 (WHO III)	235 (16.07%)	188 (12.86%)	40 (2.74%)
G4 (WHO IV)	131 (8.96%)	216 (14.77%)	106 (7.25%)
IDH_status			
Mutant	378 (25.85%)	308 (21.07%)	109 (7.46%)
Wildtype	204 (13.95%)	278 (19.02%)	139 (9.51%)
NA	7 (0.48%)	38 (2.60%)	1 (0.07%)
1p9q_status			
Codel	147 (10.05%)	135 (9.23%)	14 (0.96%)
Non_codel	437 (29.89%)	429 (29.34%)	49 (3.35%)
NA	5 (0.34%)	60 (4.10%)	186 (12.72%)
MGMT_status			
Methylated	422 (28.86%)	286 (19.56%)	80 (5.47%)
Unmethylated	140 (9.58%)	253 (17.31%)	161 (11.01%)
NA	27 (1.85%)	85 (5.81%)	8 (0.55%)
Risk			
Low	418 (28.59%)	414 (28.32%)	180 (12.31%)
High	171 (11.70%)	210 (14.36%)	69 (4.72%)

NA represented the missing value.

### Construction and Validation of the Risk Signature

Necroptosis-related genes were obtained from the GSEA website (https://www.gsea-msigdb.org/) using the keyword necroptosis. The necroptosis genes were successively filtered using univariate Cox regression analysis, least absolute shrinkage, selection operator (LASSO) Cox regression analysis, and multivariate Cox regression analysis to determine the risk genes and build the risk model. The risk signature was constructed based on the expression levels of critical necroptosis genes and their coefficients. The risk score was calculated using the following formulas:


$$Risk\;Score=\sum_{i=1}^{4}\left(\beta_{i}*Exp_{i}\right)$$

In this formula, Exp and β represent the expression levels and coefficients of each critical gene, respectively. We used the Kaplan–Meier survival curve in the TCGA cohort to define the optimal cutoff risk score. Based on the optimal cutoff value, glioma patients from the TCGA cohort were split into low and high-risk score subgroups. Kaplan–Meier survival curves and receiver operating characteristic (ROC) curves were used to detect the efficiency of the risk signature for predicting the survival of primary glioma patients. Two CGGA cohorts were used to verify the feasibility of the necroptosis risk signature.

### Independent Prognostic Analysis and Construction of the Nomogram

Univariate and multivariate analyses were performed to investigate whether the risk signature can serve as a biomarker to evaluate the prognosis of glioma patients. Subsequently, we explored the relation of the risk signature to various clinical traits using stratified analysis. We also developed a nomogram based on the risk score and identified clinical characteristics with prognostic significance based on univariate Cox analysis. The nomogram performance was evaluated by the calibration curve and ROC curves at 1, 3, and 5 years.

### Functional Enrichment Analysis and Comprehensive Analysis of the Tumor Microenvironment and Immune Cell Infiltration

To illuminate the difference of enrichment in the high-risk group compared with the low-risk group, we used GSEA to determine the results of Gene Ontology (GO) and Kyoto Encyclopedia of Genes and Genomes (KEGG) enrichment. Based on the enrichment results associated with the immune response, we evaluated the tumor microenvironment (TME) of glioma patients in the TCGA cohort using the “estimate” R package. Subsequently, the absolute infiltration fraction of 22 immune cells of glioma patients from the TCGA dataset was calculated using the online website CIBERSORTx (https://cibersortx.stanford.edu/). From the article “The Immune Landscape of Cancer” (19), we obtained information on the immune subtype of glioma patients in the TCGA database and investigated the relationship with the risk score. Finally, we downloaded the results of the correlation of the risk genes RIPK1, RIPK3, FAS, and FADD with immune cells in LGG and GBM from the TIMER database(https://cistrome.shinyapps.io/timer/).

### Therapeutic Response Prediction

The infiltration of immunosuppressive cancer-associated fibroblasts (CAFs) and myeloid-derived suppressor cells (MDSCs) in glioma patients from the TCGA cohort was calculated using the Tumor Immune Dysfunction and Exclusion (TIDE) database (http://tide.dfci.harvard.edu/) (20). We also obtained the predicted response of glioma patients for predicting immune checkpoint inhibitor (ICI) therapy.

### Statistical Analysis

The bioinformatic analyses were completed in the R (version 4.1.1) programming environment. The difference between the two groups was determined using the Wilcoxon test. Spearman’s test was adopted for correlation analyses. The KM analysis and Cox regression analysis were performed using the R packages “Survival” and “Survminer”.

## Results

### Construction and External Validation of the Risk Signature

**Figure 1:**
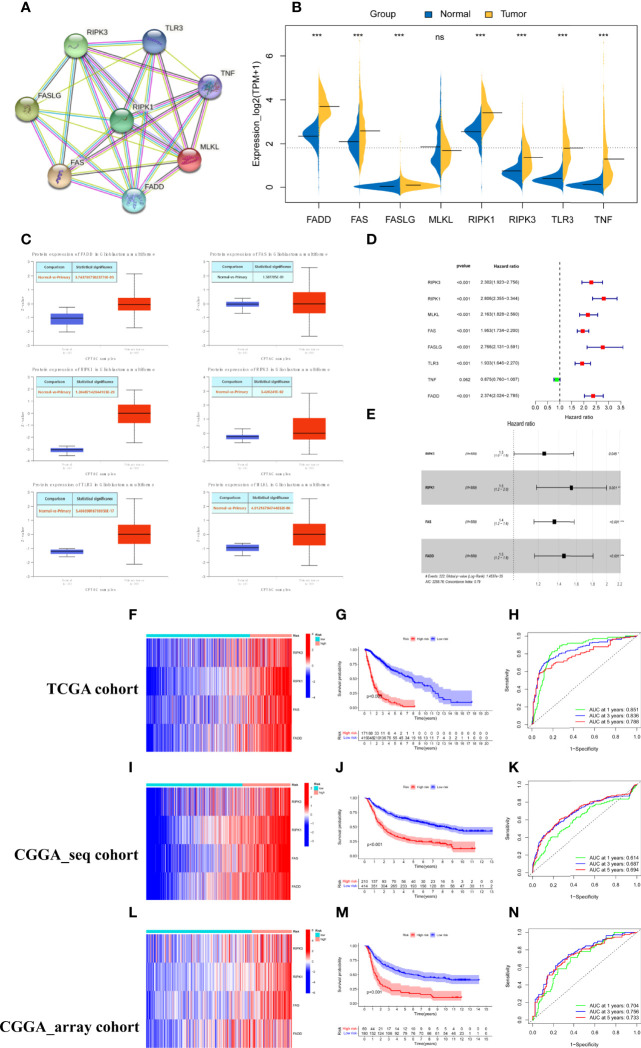
Construction and validation of the necroptosis gene signature. **(A)** PPI network of necroptosis genes. **(B)** The expression levels of 8 necroptosis genes in the primary gliomas and normal cerebral cortex tissues (* represented p < 0.05, * represented p < 0.01**,* represented p < 0.001 and ns was no statistical significance). **(C)** The protein expression levels of necroptosis genes in glioblastoma and normal brain tissues from the UALCAN database. **(D)** Screened 7 prognostic necroptosis genes by univariate cox regression analysis with a criterion of p < 0.05. **(E)** Identified 4 genes to construct the risk model using multivariate cox analysis. **(F, I, L)** Heatmap of the expression of risk genes in the training and two test cohorts. **(G, J, M)** Kaplan–Meier survival curves for comparison of survival rates between different risk populations in the TCGA cohort and validated using CGGA cohorts. **(H, K, N)** ROC curves of the risk signature for predicting prognosis at 1, 3, and 5 years in the training and two test sets.

We constructed a protein–protein interaction (PPI) network to reveal the interactions among the 8 necroptosis-related genes (**Figure 1A**). The expression levels of the 8 necroptosis genes were compared between the 589 primary glioma patients and 207 normal cerebral cortex tissues from the TCGA and GTEX datasets. As shown by the bean plot, 7 genes (RIPK1, RIPK3, FAS, FADD, FASLG, TLR3, and TNF) were upregulated in the glioma tissues with p <0.001 (**Figure 1B**). Additionally, we compared the protein expression levels of the genes between normal and glioma tissues. The results downloaded from the UALCAN database demonstrated that most necroptosis genes had higher protein expression levels in gliomas (**Figure 1C**).

To identify the critical risk genes for constructing the risk model, 7 prognostic necroptosis-related genes were screened by univariate Cox regression analysis with a criterion of p <0.05 and used as candidate genes for the next step of the analysis (**Figure 1D**). Then, the candidate genes were filtered using LASSO regression analysis, an algorithm that minimized the risk of overfitting. Finally, 4 necroptosis genes, RIPK1, RIPK3, FAS, and FADD, were determined to be critical genes for building the risk model by multivariate regression Cox analysis. All four genes were associated with a poor prognosis (hazard ratio [HR] >1, **Figure 1E**). The risk scores of glioma patients were calculated based on the coefficient and expression levels of each gene and the formula was as follows: risk score = 0.431 * RIPK1 +0.227 * RIPK3 + 0.302 * FAS +0.373 *FADD. To obtain the best effective grouping, we used the “surv_cutpoint” algorithm to calculate the optimal cutoff value of glioma patients in the training set and split the glioma patients from the TCGA cohort into low and high-risk score subgroups. To inspect the consequences of grouping, the principal component analysis (PCA) was completed and the results showed that the glioma population was clearly separated into two subgroups (**Supplementary Figure 1A**). The heatmap indicated that patients with high-risk scores had higher expression levels of the risk genes (**Figure 1F**). The Kaplan–Meier survival curve and the survival status scatter plot indicated that populations with high-risk scores had a poor overall survival outcome compared with that of the low-risk group in the TCGA cohort (**Figure 1G** and **Supplementary Figure 1D**). Additionally, the sensitivity and specificity of the risk scores to predict the overall survival (OS) of patients were assessed by ROC curves. The areas under the curve (AUCs) of the risk score at 1, 3, and 5 years were 0.851, 0.836, and 0.788, respectively (**Figure 1H**). Based on the training cohort optimal cutoff risk score, patients from the two CGGA cohorts were divided into two subgroups to verify the accuracy of the risk signature for predicting survival. Similar to the training set, significant differences in the distribution (**Supplementary Figures 1B, C**) and survival status (**Supplementary Figures 1E, F**) of patients were noted between the low- and high-risk subgroups in the test datasets. Similar consequences were obtained while the expression heatmap (**Figures 1I, L**), KM survival curve (**Figures 1J, M**), and time-dependent ROC analyses (**Figures 1K, N**) were obtained using the two CGGA cohorts. In conclusion, the four-necroptosis gene signature can accurately predict the overall survival of glioma patients.

### Independent Prognostic Values of the Risk Signature and Its Relation to Clinical Characteristics

**Figure 2:**
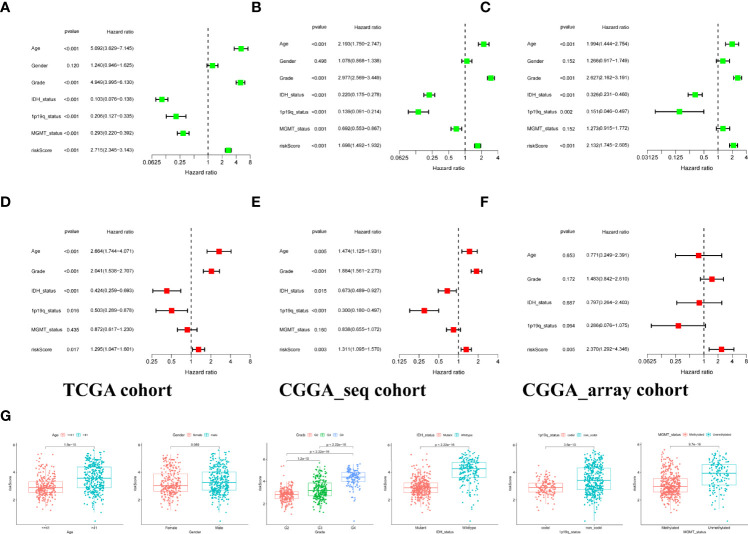
Independent prognostic analysis of risk signature. **(A–C)** Identification of the prognostic values of clinical characteristics and risk signature *via* univariate cox regression analysis. **(D–F)**. A comprehensive evaluation of independent prognostic value after offsetting the effects of other factors by multivariate Cox regression analysis. **(G)** Stratified analysis of risk scores with various clinicopathological parameters in the training set.

The association of risk and clinicopathological parameters with OS was evaluated using univariate Cox regression and multivariate Cox analysis. As shown by the forest plots of univariate analysis, similar to various clinical features including age, tumor grade, IDH mutation status, 1p19q codeletion, and MGMT methylation, the risk score could serve as an independent prognostic factor (p<0.001, HR:2.715, 1.698, 2.132, **Figures 2A–C**). Subsequently, we further performed multivariate Cox analysis to counterweigh the effects of other factors and found that the risk score remained statistically significant (p<0.05, HR: 1.295, 1.311, 2.370, **Figures 2D–F**). We also compared the risk score between glioma patients with different clinical characteristics in the TCGA set and found that the population classified as the high-risk group had higher risk scores (**Figure 2G**).

### Construction of the Nomogram for Individualized Prognostic Prediction

**Figure 3:**
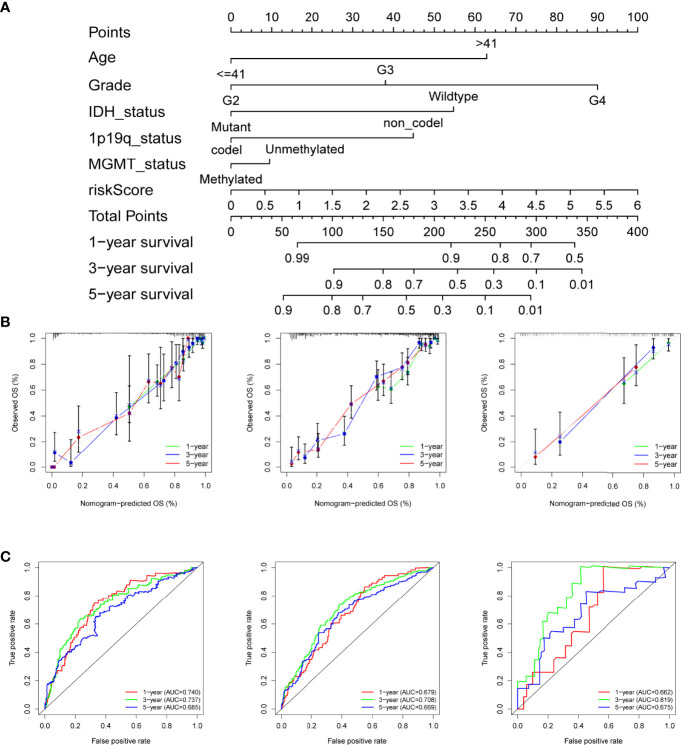
Construction of the nomogram for predicting overall survival. **(A)** Nomogram plot for predicting the survival rates of glioma patients at 1, 3, and 5 years in the TCGA cohort. **(B)** The calibration curves of the nomogram for assessing the consistency of actual and predicting OS at 1-, 3-, and 5-year in the training and the two test cohorts. **(C)** ROC curves of nomogram at 1, 3, and 5 years in the different datasets respectively.

Given the importance of the risk score and various clinical features, a nomogram was built that combined the risk score and prognostic clinical traits screened by univariate Cox analysis to maximize the efficiency for predicting individual prognosis (**Figure 3A**). As shown by the plot (**Figure 3B**), the 1-, 3-, and 5-year calibration curves for the nomogram demonstrated that the predicted nomogram was capable of forecasting the survival time of patients from the training set accurately and obtained similar results in the two test sets. In addition, we utilized ROC to evaluate the sensitivity of the nomogram model. The AUCs of the nomogram at 1, 3, and 5 years were 0.740, 0.737, and 0.685, respectively, in the TCGA cohort and validated by the two CGGA cohorts with all AUCs values greater than 0.65 (**Figure 3C**), indicating that the nomogram could distinguish patients with good or poor prognoses.

### Functional Enrichment Analysis Based on the Risk Signature

**Figure 4:**
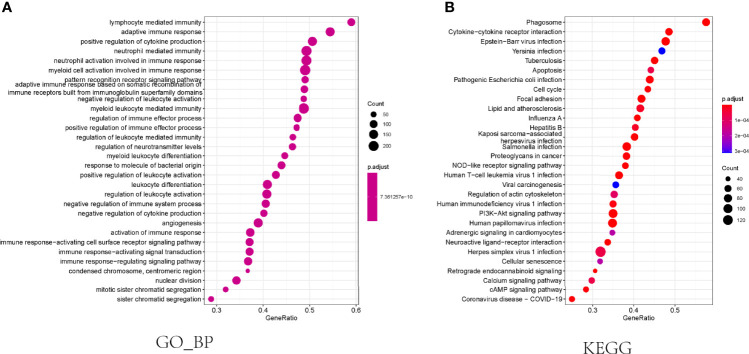
Functional enrichment analysis in the TCGA cohort. **(A)** The results of biological progress enrichment analysis of differently expressed genes between high- and low-risk subgroups. **(B)** Bubble graph for KEGG enrichment analysis.

To determine the difference in the biological processes and pathways between the subgroups categorized by the necroptosis gene model, we utilized the R package “limma” to identify the differentially expressed genes and defined the log2-fold change (logFC) as the median gene expression level of the high-risk subgroup minus the value of the opposite subgroup in the TCGA cohort. Subsequently, GO and KEGG enrichment analyses between the low- and high-risk groups were performed using the GSEA algorithm. The results showed that these differentially expressed genes were enriched in the biological process of multiple immune cell-mediated immune response, regulation of cytokine production, multiple immune cells activation, angiogenesis (**Figure 4A**), and pathways related to cancer or immune response, including cytokine−cytokine receptor interaction, apoptosis, cell cycle, PI3K−Akt signaling pathway, NOD−like receptor signaling pathway, and cellular senescence(**Figure 4B**).

### Correlation of Risk Score With Immune Activity

**Figure 5:**
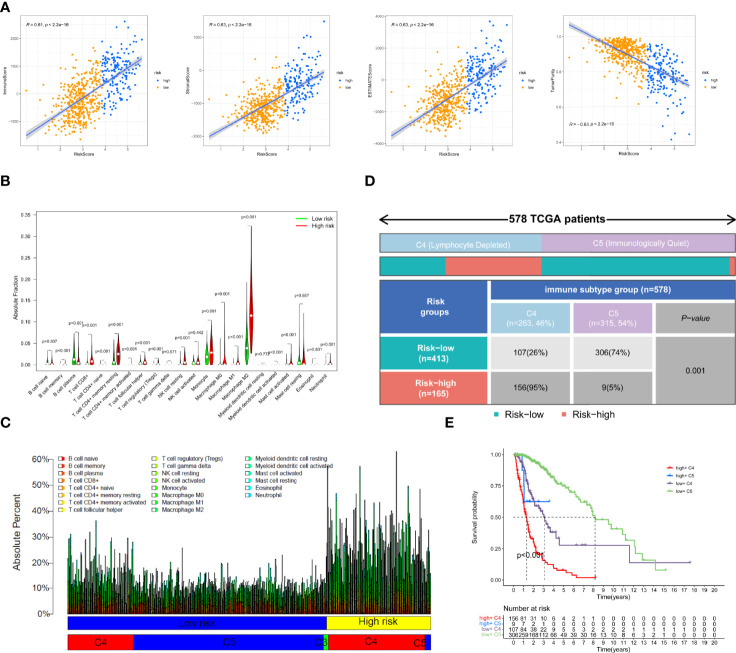
Tumor microenvironment and immune cell infiltration analysis in the TCGA cohort. **(A)**The correlation of immune scores, stromal scores, ESTIMATE scores, and tumor purity with risk scores. **(B)** Comparison of the degree of infiltration of 22 immune cells in the different risk groups. **(C)** The absolute fraction of immune cells between different subgroups in the TCGA glioma patients. **(D)** Heatmap and table showing the distribution of immune subtype between the two risk groups in the TCGA cohort. **(E)** Comparison of the survival difference between groupings after combining risk signature and immune subtypes by Kaplan–Meier survival curves.

To explore the association of necroptosis with immune activities, we calculated the tumor microenvironment scores using the R package “ESTIMATE”. The results of correlation scatter plots revealed that the risk score was positively correlated with the immune score, stromal score, and ESTIMATE score (R=0.61, 0.63, and 0.63, respectively), but negatively correlated with the tumor purity (R= -0.63, **Figure 5A**). Additionally, we analyzed the infiltration fraction of 22 immune cells using the CIBERSORTx algorithm. The violin plot indicated the difference in 22 immune cells between the low- and high-risk groups. Multiple immune cells, including Macrophages (M0, M1, M2), regulatory T cells (Tregs), neutrophils, monocytes, resting NK cells, and CD8+ T cells were highly infiltrated in the high-risk group, whereas only plasma B cells were enriched in the low-risk group (**Figure 5B**). In addition, we visualized the absolute infiltration fraction of 22 immune cells using a percentage histogram, which suggested that the glioma population with high expression of necroptosis genes exhibited a greater level of total immune cell infiltration, which is similar to the results of TME analysis. We also combined the immune subtype information of glioma patients from the TCGA cohort to further evaluate the difference in immune activity between the low- and high-risk subgroups. The lymphocyte-depleted immune subtype (C4) had a higher infiltration fraction than the immunologically quiet subtype (C5) (**Figure 5C**). Because the proportion of the inflammatory immune subtype (C3) in the TCGA glioma cohort was very low, we excluded these C3 subtype populations for further analysis. In addition, we found that the low-risk group was mainly dominated by the C5 subtype (74%), whereas the high-risk group was dominated by the C4 subtype (95%, **Figure 5C**). A significant correlation was noted between the risk grouping and the immunotype as assessed using Fisher’s test (p=0.001, **Figure 5D**). The Kaplan–Meier survival curve revealed that patients with “low-risk + C5” subtypes had the best prognosis, while those with “high-risk + C4” subtypes had the worst prognosis (**Figure 5E**). Additionally, we downloaded the results of the correlation analysis of the risk genes RIPK1, RIPK3, FAS, and FADD with multiple immune cells using the online website Timer database to validate the accuracy of our immune analysis. Based on this analysis, the necroptosis genes were highly positively correlated with the infiltration of lymphocytes, neutrophils, macrophages, and dendritic cells (DC) (**Supplementary Figures 2A–D**). The correlation suggested the accuracy of our immune analysis.

### Immune Analysis by TIDE Database

**Figure 6:**
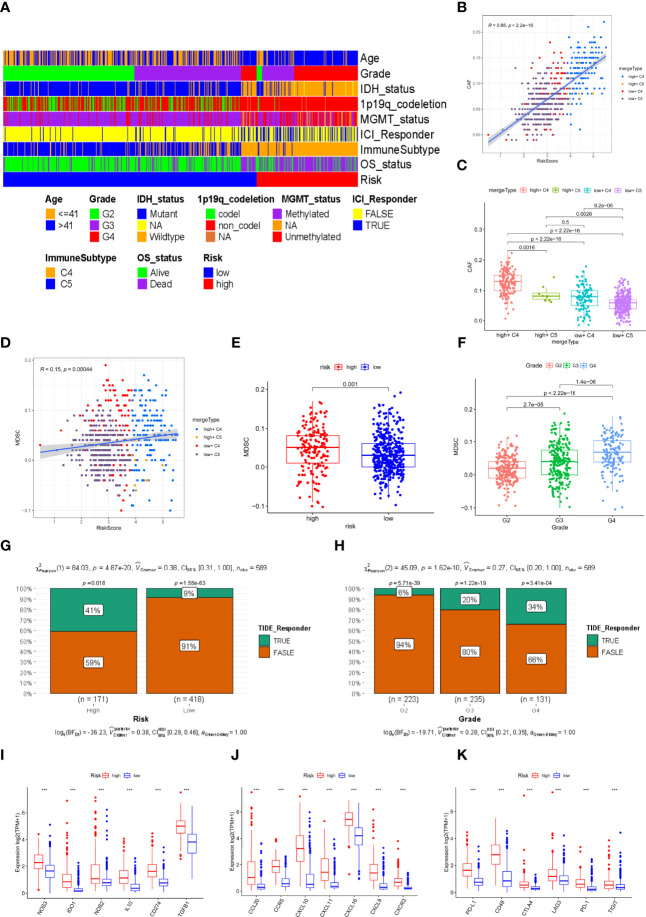
Prediction of immune therapy and the correlation of immunosuppressive cell with risk signature in the TCGA cohort. **(A)**The distribution of multiple clinical characteristics, immune subtypes, and survival status in the high- and low-risk groupings. **(B, D)** The correlation of the risk score with CAFs and MDSCs. **(C)** The difference of infiltration fraction of CAFs in the different subtypes. **(E, F)** The infiltration fractions of MDSCs in the different risk groups and tumor grades. **(G, H)** Distribution of the predicted responsiveness of glioma patients for the ICI therapy in the different risk groups and tumor grades by the TIDE database. **(I–K)** The different expression levels of immunosuppressive cytokines, tumor-associated chemokines, and immune checkpoints related genes (*** represent p < 0.001).

To investigate the infiltration of CAFs and MDSCs in glioma patients in the TCGA cohort, we used the TIDE database. Furthermore, we constructed a heatmap to summarize and reveal the distribution of various traits in the risk signature. As shown in the plot, we found that almost all patients from the high-risk subgroup had the C4 immune subtype, tumor grade 3 or 4, and high mortality, with a higher proportion of poor prognostic factors such as IDH wild type, 1p19q non-codeletion, and MGMT unmethylation. The opposite trends were noted in the populations with a low risk. (**Figure 6A**). CAFs were significantly positively related to the risk score (R=0.65, **Figure 6B**). Comparing the infiltration score of CAFs between different risk groupings and immune subtypes, we found that CAFs were more enriched in the populations with high-risk scores and C4 immune subtypes (**Figure 6C**). We also visualized the correlation of MDSCs with risk scores and different MDSCs between different groupings and tumor grades. Unexpectedly, the results indicated that MDSCs were weakly correlated with risk scores (**Figure 6D**) but they had a higher infiltration fraction in the high-risk subgroup (**Figure 6E**) and tumor grade 4 (**Figure 6F**). Additionally, we assessed the difference of CAF and MDSC infiltration fractions in patients with different clinical characteristics. Unexpectedly, the infiltration score of MDSCs in the 1p19q codeletion group was higher than in the non-codeletion group and CAFs were highly enriched in the 1p19q non-codeletion population. The remaining results showed that MDSCs and CAFs were less infiltrated in patients with favorable clinical characteristics, such as young age, IDH mutant, and MGMT methylation (**Supplementary Figures 3A, B**). Since TCGA patients have not received ICI treatment, we aimed to calculate the response rate of glioma patients to immune checkpoint inhibitors by the TIDE algorithm. The predicted results suggested that individuals with higher risk scores (**Figure 6G**) or tumor grade 4 (**Figure 6H**) obtained a better response compared to other group patients. Finally, we compared the expression levels of immunosuppressive cytokine genes in the low and high-risk groups, revealing that these genes were highly enriched in the high-risk group (**Figure 6I**). Additionally, tumor-associated chemokines (**Figure 6J**) and immune checkpoint genes (**Figure 6K**) were enriched to the same levels as immunosuppressive cytokines.

## Discussion

As a genetic disease, tumors are generally thought to be the outcome of a succession of genetic abnormalities that activate oncogenes and inactivate tumor suppressor genes, resulting in aberrant cell proliferation. These extensive genetic changes are important factors affecting the development of tumors, so the identification of potential tumor-related biomarkers will help researchers further elucidate the molecular characteristics of tumors. Necroptosis is a novel type of cell death discovered in recent years that has facilitated the recognition of the composition of programmed cell death. A variety of analyses have shown that necroptosis is a promising therapeutic strategy for addressing apoptosis resistance in tumors (11). Necroptosis has dual roles in promoting and inhibiting tumor formation (10, 21). Necroptosis can suppress tumor growth by promoting dendritic cells (DCs) to release IL-12 (22) and activate cytotoxic T cells (23, 24). Conversely, necroptosis genes themselves or the inflammatory response caused by necroptosis can promote tumor progression by fostering angiogenesis, promoting cancer cell proliferation, accelerating cancer metastasis, and promoting genomic instability (11). However, the effect of necroptosis in glioma has not been completely elucidated.

In this study, we first clarified the expression levels of necroptosis genes in normal brain tissue and tumor tissue. Interestingly, our data showed that necroptosis genes, except with the expression of MLKL, were significantly upregulated in glioma. Univariate Cox regression analysis revealed that necroptosis genes were associated with a worse prognosis in the glioma population, suggesting that necroptosis may play a cancer-promoting role in glioma. It was consistent with previous studies, Park, Hatanpaa et al. showed that RIPK1, the key gene of necroptosis, was commonly overexpressed in glioblastoma and positively correlated with worse survival (25) and RIPK3 was also identified as a negative prognostic biomarker in low-grade glioma (26). Additionally, studies showed that necroptosis genes were highly enriched in other tumors and correlated with poor prognosis. For instance, RIPK1 was highly enriched in lung cancer and pancreatic ductal carcinoma and accelerated the development and metastasis of the tumor (18, 27). Four necroptosis genes, RIPK1, RIPK3, FAS, and FADD, were identified by LASSO and multivariate Cox analysis and used to construct a risk signature. We performed a series of analyses to estimate the accuracy and specificity of the necroptosis genes signature for predicting prognosis and validated it using CGGA datasets. The results demonstrated that the necroptosis-related gene signature exhibited good performance in assessing the prognosis of glioma patients. Subsequently, functional enrichment was performed to explore the possible biological functions of necroptosis. The GO and KEGG results suggest that necroptosis genes may be related to the immune response and angiogenesis biological progressions and PI3K-Akt signaling pathways. In fact, the key genes of necroptosis have been confirmed to be related to immune activity and angiogenesis. Ueta, T et al. found that RIPK1 was highly expressed in infiltrating M2 macrophages and mediated pathological angiogenesis (28) and RIPK3 was reported that it could modulate the growth factor receptor of endothelial cells to support angiogenesis (29).

Based on the results of functional enrichment analysis, we further performed immune analysis to assess the relationship between necroptosis and the immune microenvironment in glioma. The TME and CIBERSORTx absolute infiltration scores clarified that the patients from the high-risk subgroup obtained more immune and stromal cell infiltration. In particular, M2 macrophages were the most abundant among these immune cells (30). Combining the results of the immune subtype of all TCGA glioma patients from previous research (19), we found that the high-risk group was dominated by the C4 subtype, which was mainly characterized by lymphatic depletion. In contrast, the immune quiet subtype (C5) accounted for a higher proportion of individuals in the low-risk group and had a longer survival time. In addition, we determined that necroptosis probably affected the outcome of immunophenotyping by Fisher’s test. Based on immune analysis results, we reasonably inferred that necroptosis can boost immune activity. Overall, it appeared to be an immunosuppressive response, which did not improve, but worsen, the prognosis of glioma patients. Additionally, our data on the distribution of clinical characteristics in glioma patients showed that those who obtained high-risk scores had more unfavorable prognostic clinical traits. Research demonstrated that the C4 subtype gives it the worst prognosis of the constituent tumor and shows a composite signal reflecting the dominance of macrophages, low lymphocyte infiltration, and high content of M2 macrophages, which is consistent with immunosuppressive TME and its poor prognosis (19). Importantly, previous research has found that individuals with clinical features including IDH mutation, 1p19q codeletion, and MGMT methylation, were more likely to be in the C5 subtype (31) and possess a better immune microenvironment (32), which decreased leukocyte chemotaxis and infiltrated tumor-related immune cells (33). In addition, concerning the subtype of lymphatic depletion, we found that the high-risk group dominated by the C4 subtype had a higher proportion of T lymphocytes. We believed that lymphocyte depletion may refer to the reduction of the number of lymphocytes, and conversely, it means the functional depletion of T cells caused by the loss of their normal function. Studies have shown that T cell exhaustion was particularly serious in gliomas and was characterized by up-regulation of multiple immune checkpoints and slow T cell response (34, 35). To some extent, this finding explained why patients in the group with high necroptosis genes or C4 immune subtype had a worse prognosis and why these genes played a cancer-promoting role in glioma.

Finally, the results of predicting the response to immune checkpoint inhibitor (ICI) therapy using the TIDE database showed that necroptosis was strongly correlated with CAFs and weakly correlated with MDSCs, but the two immunosuppressive cells were enriched in the high risk and C4subtype populations. These findings indicate that necroptosis may attract the infiltration of these immunosuppressive cells in regulating the immune environment, thereby promoting glioma cell growth and immune escape. Jayakumar demonstrated that the necroptosis gene RIPK3 can induce inflammation through intermediate MDSCs to promote intestinal tumor growth (36). This result effectively explains the phenomenon of the high-risk population having a higher immune infiltration score and a poorer prognosis. Although previous experimental research revealed that necroptosis kills cancer cells and exerts antitumor effects in glioma (37–40), necroptosis did not improve the glioma patients’ prognosis and some research showed that necroptosis-related genes were unfavorable prognostic markers in gliomas (26, 41). We believed that necroptosis exerted an anti-tumor effect to promote glioma cells’ death, conversely, tumor cells released a variety of immunosuppressive cytokines and chemokines and necrotic tumor cells also attracted a variety of immune cells infiltrating. We can see that the high-risk group had more immune suppressive cells, such as Tregs, M2 macrophages, MDSCs, and TAMs, releasing a variety of immunosuppressive cytokines and chemokines. Chronic inflammation due to long-term infiltration further aggravates the tumor suppressor microenvironment and promotes tumor growth (42, 43). In addition, research has suggested that the induced chemokines play a negative role in the anticancer effect. For example, RIP1/RIP3 induces the release of CXCL1, which causes immunosuppression and promotes pancreatic oncogenesis (18). Although the high-risk group has a considerable proportion of resting memory CD4+ T cells, studies have shown that tumor cells can weaken T cell reactivity and antigen processing capacity, so that T cells cannot effectively control tumor growth (44). Continuous expression of PD1 could lead to T cell exhaustion and exhausted T cells lost their normal functions, a mechanism that promoted tumor cells to escape immune killing (45, 46). Immune checkpoint inhibitors killed tumor cells by inhibiting PD1 expression and further promoting T cell activation. Interestingly, the response to ICI therapy, predicted by TIDE, showed that high-risk groups and high-grade populations may have a greater chance of benefiting from ICI therapy, whereas the heatmap indicated that the population with high necroptosis activity was mainly immunotype C4 and tumor grades 4 and 3. The necroptosis-related genes signature was expected to be a potential marker for predicting and guiding ICI therapy. We found that the high-risk group has a higher proportion of T cell infiltration and T cells were the main effector cells of the immune response and could obtain more activated effector T cells in ICI therapy to kill tumor cells (47). It was a possible explanation for the higher immunotherapy response rate of patients with higher risk scores and tumor grades. However, the past clinical trials have shown that glioblastoma patients did not have such a high response rate to ICI therapy. ICI therapy was affected by various factors, such as the lack of inflammatory response in the tumor microenvironment, the inactivation of T lymphocytes, and the lack of sufficient or suitable antigens and other mechanisms, all of which affected the effect of ICI therapy (48). Significantly, the TIDE database only recommended the prediction of the response of non-small cell lung cancer and melanoma to ICI therapy. There is a certain degree of uncertainty in predicting the response of gliomas to ICI therapy through TIDE algorithm and further research is needed to determine the accuracy of TIDE results.

Our study preliminarily explored the value of necroptosis genes in glioma and this information provides a theoretical basis for future research. As this was a bioinformatic study, there are still certain limitations. The data were obtained from public databases. This study lacks sufficient experimental validation and mechanistic research and our model needs to be validated by subsequent basic experiments and clinical prediction research. Finally, the role of necroptosis in gliomas must be further investigated.

## Conclusion

In conclusion, based on comprehensive bioinformatics analysis, we identified a necroptosis gene signature that can predict prognosis in glioma patients and assessed the association of this signature with the immune microenvironment and response to ICI therapy. We think our research will provide valuable advice on follow-up studies.

## Data Availability Statement

The original contributions presented in the study are included in the article/**Supplementary Material**. Further inquiries can be directed to the corresponding author.

## Author Contributions

ZZ and JX contributed to this research equally. ZZ, JX, and YC contributed to the conceptualization and investigation. ZZ and JX contributed to the methodology, data analysis, visualization, and Writing-original draft. NH, JT, and PM contributed to the methodology, investigation, visualization. ZZ, JX, NH, JT, PM, and YC contributed to the Writing-review and editing. YC contributed to the funding acquisition and project administration. All authors have read and approved the submitted version.

## Funding

This study was funded by grants from the National Natural Science Foundation of China (No.81771961) and the Chongqing Natural Science Foundation of China (cstc2021jcyj-msxmX0036).

## Conflict of Interest

The authors declare that the research was conducted in the absence of any commercial or financial relationships that could be construed as a potential conflict of interest.

## Publisher’s Note

All claims expressed in this article are solely those of the authors and do not necessarily represent those of their affiliated organizations, or those of the publisher, the editors and the reviewers. Any product that may be evaluated in this article, or claim that may be made by its manufacturer, is not guaranteed or endorsed by the publisher.
