# Supplementary figures and images for: Clinical and Biological Significance of a Necroptosis-Related Gene Signature in Glioma
Source: Front Oncol. 2022 Jun 2;12:855434. doi: 10.3389/fonc.2022.855434 (PMC9201102; doi:10.3389/fonc.2022.855434)

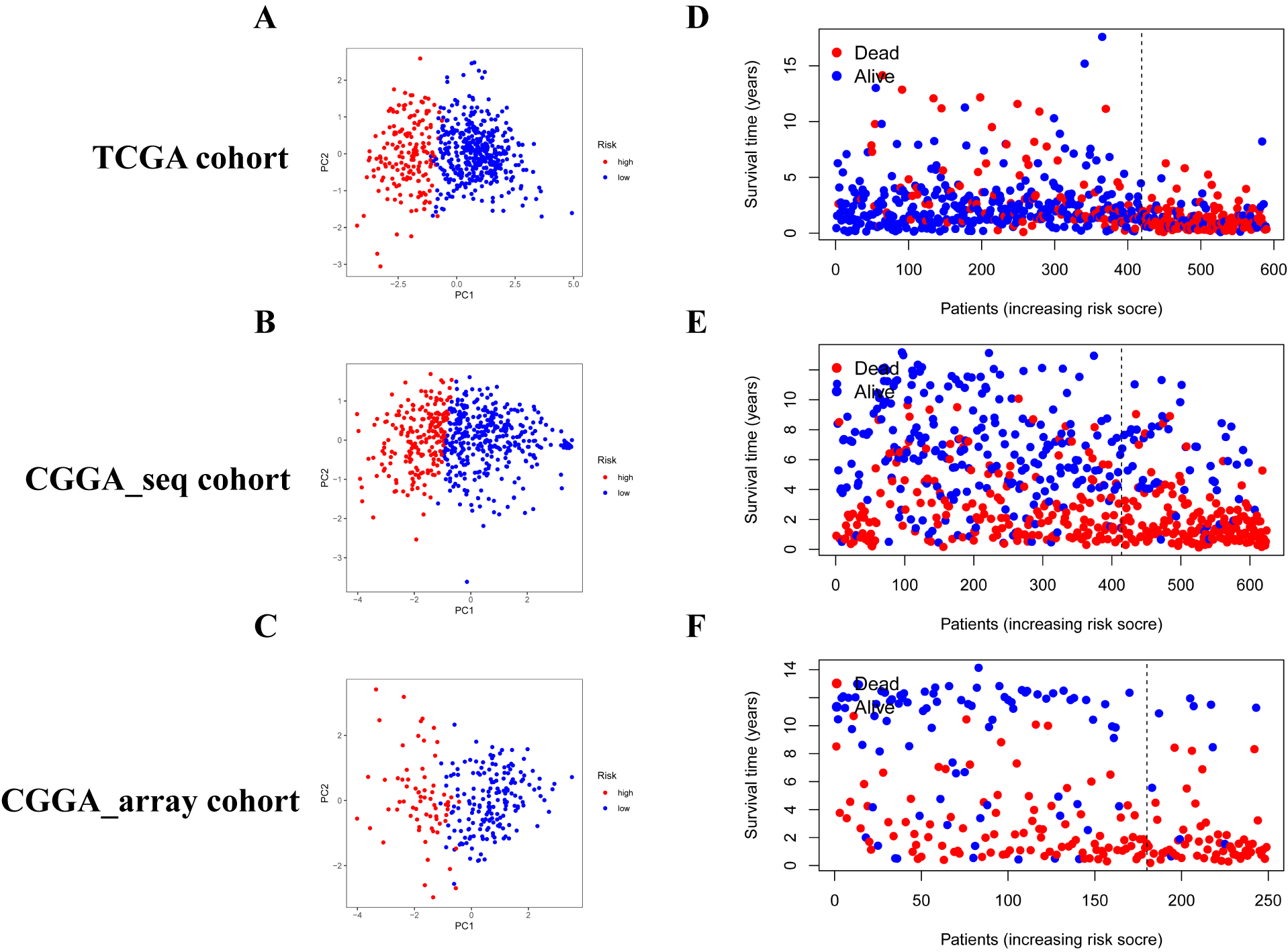

Supplement: Supplementary file 1 [file Image_1.tif]

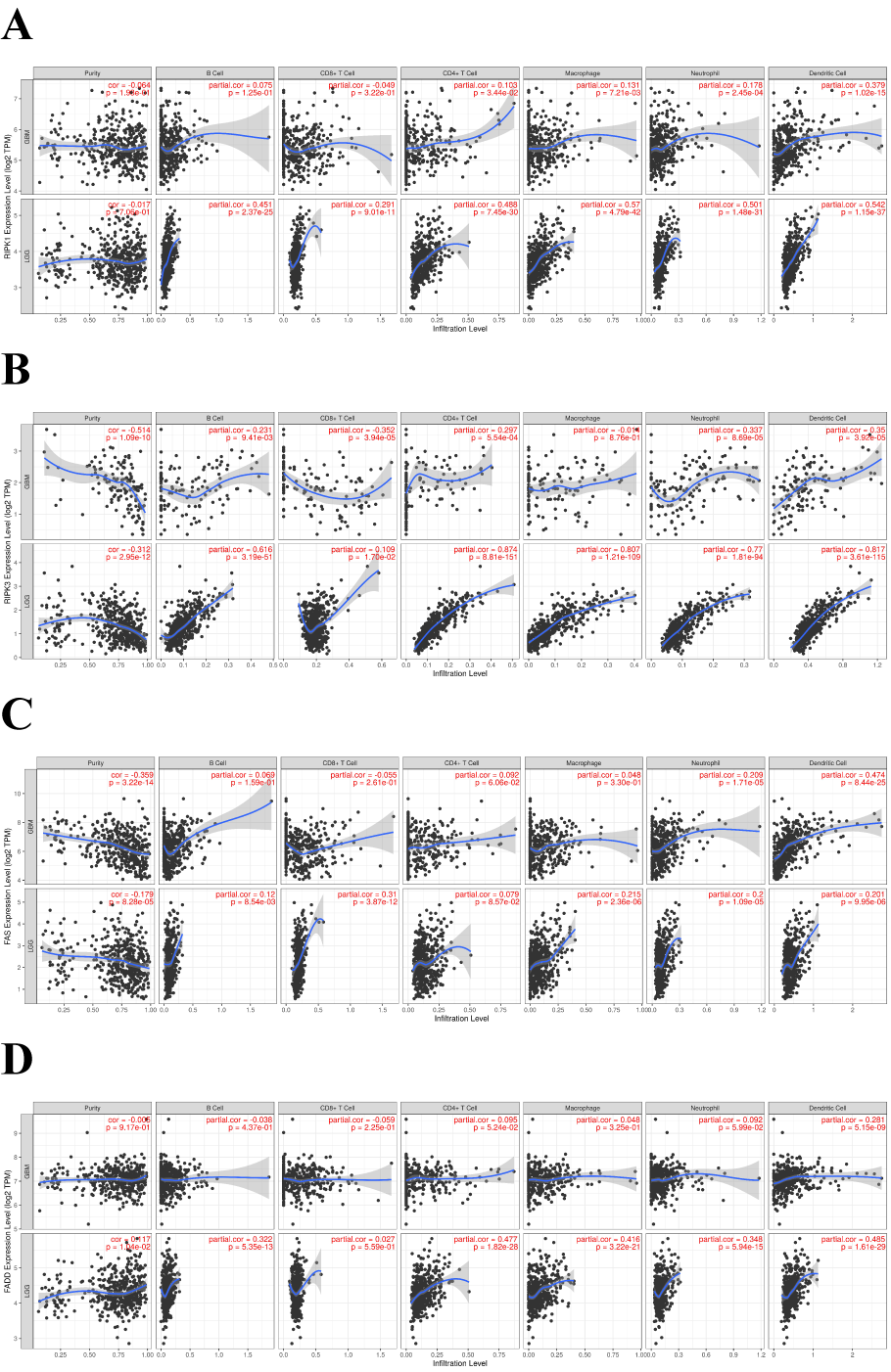

Supplement: Supplementary file 2 [file Image_2.tif]

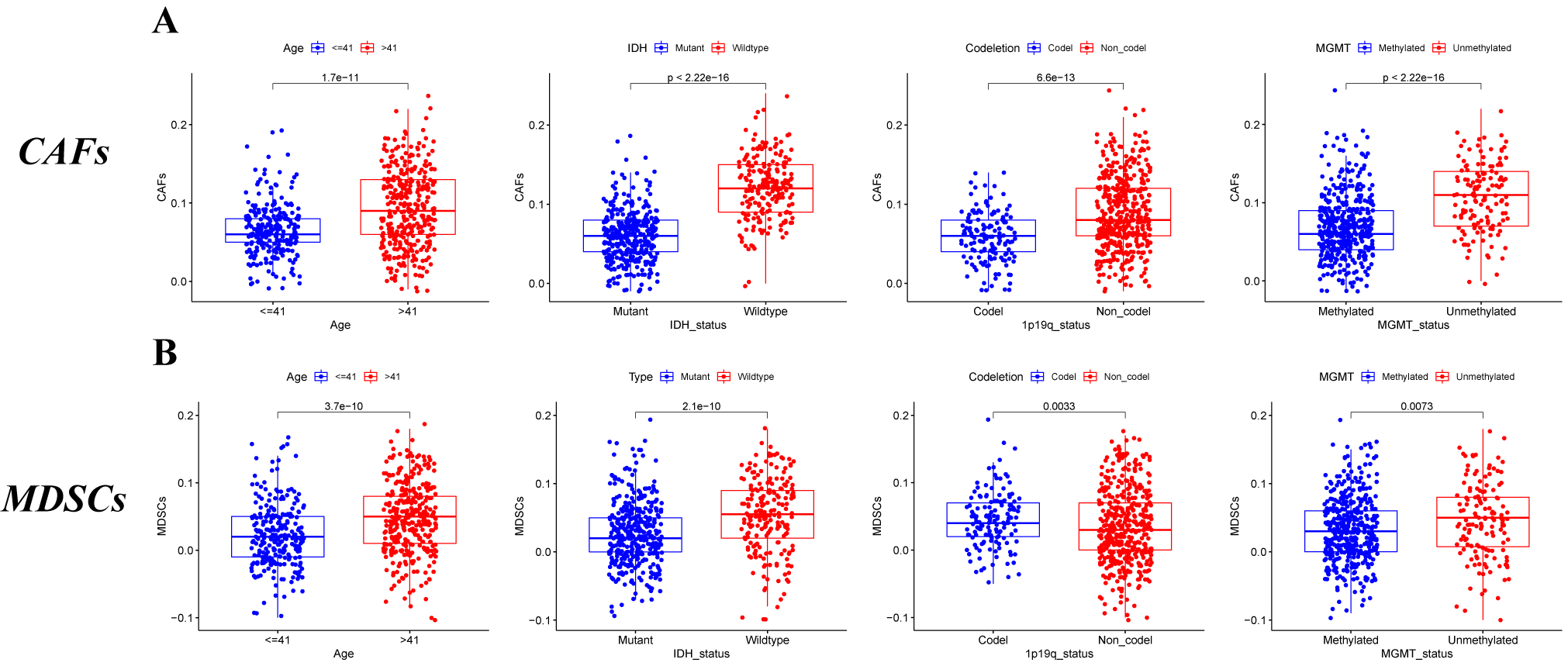

Supplement: Supplementary file 3 [file Image_3.tif]
